# Where environment and malaria intersect: Exploring the spatio-temporal footprints of *Plasmodium falciparum* in Tanzania

**DOI:** 10.1371/journal.pone.0321200

**Published:** 2025-05-27

**Authors:** Kamaldeen Mohammed, Satveer Dhillon, Cornelius K.A. Pienaah, Isaac Luginaah, Eva-Maria Knoll, Gwyn Campbell, Herbert Hambati

**Affiliations:** 1 Department of Geography and Environment, University of Western Ontario, London, Ontario, Canada; 2 Institute for Social Anthropology, Austrian Academy of Sciences, Vienna, Austria; 3 Department of History and Classical Studies, McGill University, Montreal, Quebec, Canada; 4 Population Studies and Research Centre, College of Social Sciences, University of Dar es Salaam, Dar es Salaam, Tanzania; The University of Dodoma, UNITED REPUBLIC OF TANZANIA

## Abstract

Malaria remains a public health crisis in Tanzania, with persistent morbidities and mortalities. Malaria etiology is multifactorial, with environmental factors playing a vital role in mosquito development and malaria transmission. In Tanzania and most of Sub-Saharan Africa, the *Plasmodium falciparum* parasite remains the most prevalent and virulent malaria parasite. Using data from the Tanzania Demographic and Health Surveys and spatio-temporal analysis, we explore the environmental determinants of *P. falciparum* across different regions in Tanzania over the last 2 decades. The hotspots analysis showed that the Kigoma and Kagera regions in the north-west of Tanzania as well as the Lindi and Mtwara regions in southern Tanzania were consistently hotspots of *P. falciparum* malaria from 2000 to 2020. Our findings also reveal and reinforce the role of environmental factors in mediating malaria epidemiology in Tanzania. Factors such as the use of insecticide-treated nets, population, evapotranspiration and aridity were often adversely associated with *P. falciparum* incidence. In contrast, vegetative landcover, temperature, precipitation, and the number of wet days were directly associated with *P. falciparum* in Tanzania. However, the relationship between these environmental factors and malaria prevalence varied temporally and spatially. Our findings further showed that, the two most important environmental factors that mediate *P falciparum* incidence in Tanzania over the last two decades were precipitation and aridity. Other vital predictors included the use of insecticide nets and the number of wet days. The findings provide policy pointers for targeted malaria interventions in Tanzania in the context of environmental change.

## Introduction

One of the critical public health threats, particularly in Sub-Saharan Africa (SSA), is the ongoing challenges of malaria control, despite the efforts towards reducing and eliminating the prevalence [1,2]. Indeed, SSA accounts for 95% of all global cases of malaria and 96% of all malaria deaths, with children under the age of 5 accounting for 80% of these deaths [1]. Despite, the total funding for malaria control and elimination reaching 3 billion dollars, the prevalence of malaria remains far from elimination in SSA [3]. Tanzania is among the seven countries in the World Health Organization (WHO) African Region with the highest malaria burden [4], with over 93% of the population still at risk of malaria [5]. While there has been a decline in malaria transmission recently, malaria continues to be a leading cause of morbidity and mortality in Tanzania [6]. To highlight, the WHO reports that Tanzania is one of four countries that accounts for just over half of all malaria deaths worldwide [7]. Vulnerable populations, such as pregnant women and children, are more likely to be disproportionately impacted by malaria. Malaria continues to remain responsible for up to one-fifth of deaths among pregnant women and more than one-third of deaths among children under the age of 5 [8].

There are a complex number of factors that contribute to malaria transmission. At the individual level, misconceptions surrounding bed nets and indoor residual spraying, being of lower socioeconomic status, residing in homes built of mud, and not adhering to treatment regimens are all factors that can drive malaria prevalence [6]. Also, residing further away from healthcare facilities and visiting traditional healers can lead to the persistence of malaria [5,9]. Further, in Tanzania, there is a lack of an effective malaria surveillance system, which prevents public health experts from creating targeted interventions for at-risk locations [8,10]. Environmental conditions, such as higher cropland cover, and stagnant water are also associated with increased malaria transmission in the context of Tanzania [11,12]. Evidence also indicate that malaria incidence is connected with ecological and climate variability in Tanzania [12]. For example, rainfall is associated with seasonal peaks of malaria transmission [13,14].

To exacerbate the preceding concerns, the main malaria parasite in Tanzania is the *Plasmodium Falciparum,* one of the most severe malaria parasites [15]. For example, the *Plasmodium* parasite species is considered the deadliest globally and accounts for more than 90% of the world’s malaria mortality [16–18]. The other major malaria parasites, including *Plasmodium malariae*, *Plasmodium vivax,* and *Plasmodium ovale* have low prevalence in Tanzania [15].

While the role of various environmental and climatic factors in malaria transmission has been acknowledged in the literature [19–21], a scant amount of studies have thoroughly explored the importance of geographical and temporal variations in associated environmental factors and the role of this research in the creation of malaria control and prevention programs at both local and national levels. Importantly, even within Tanzanian national boundaries climatic and ecological processes vary significantly [22]. Therefore, there is a need to consider how local-specific climatic factors impact differences in malaria prevalence, as this information will be useful for public health policy. To this end, we contribute to the literature by exploring the geographical heterogeneity of environmental correlates of malaria prevalence in Tanzania in the last two decades. Findings from this study will help provide insights into malaria control and prevention by highlighting how environmental factors and variations can be incorporated into malaria control policy planning. Further, the findings will be useful not only in Tanzania, but in other countries in SSA that are striving to achieve Sustainable Development Goal 3, with a particular focus on Target 3.3, which states “*End the epidemics of AIDS, tuberculosis, malaria and neglected tropical diseases and combat hepatitis, water-borne diseases and other communicable diseases* [23].”

### Environmental determinants of diseases

Patterns of infectious diseases have been associated with environmental changes across time and space [24]. For instance, there has been an increased focus on how climate change and urbanization can increase the prevalence of infectious diseases such as malaria, dengue fever, cholera and Lyme disease [25–27]. Hence, it is imperative to consider and utilize frameworks that help explain the underlying mechanisms of environmental factors impacting disease transmission.

One such example is the Environmental Determinants of Diseases (EnvID) framework. Made up of three interlocking components: environment, transmission, and disease [28], the foundation of this framework states that environmental processes impact the transmission cycles of infectious diseases. The environmental component has been disaggregated into distal environmental changes and proximal environmental characteristics. Distal environmental changes, deemed to be larger changes on a spatio-temporal scale, are those that impact disease transmission through multiple steps [28]. Examples include climate change, antibiotic usage, agricultural intensification, deforestation and urbanization [28]. These changes impact disease patterns through a series of casual linkages [28]. For example, climate change can impact precipitation levels, which may increase rainfall levels, creating stagnant water in certain areas, which increases the breeding and growing grounds for mosquitoes, which may carry malaria, yellow fever, dengue and West Nile Fever [29]. Proximal environmental characteristics are directly measurable components of the environment that directly impact the environment of the organisms and may directly affect the transmission cycle of an infectious disease [28]. Examples include temperature, precipitation, population density and humidity [28]. In some instances, it is not possible to distinguish between distal changes and proximal environmental characteristics, as it presents a continuum and is spatio-temporally dependent [28]. Thus, it is imperative to focus on proximal environmental factors as they are measurable, which can help in describing the relationship between environmental factors (such as rainfall and temperature) and infectious diseases (such as malaria). By establishing the relationship between these measurable factors and malaria prevalence in different regions of Tanzania, policy directions can become focused and specific, further targeting and benefiting those most at risk.

Utilizing the framework will assist in providing an overarching conceptualization to better understand the relationships between environmental factors and malaria prevalence in Tanzania. To reduce the burden of malaria in Tanzania, public health experts need to incorporate the risk factors of malaria into policy and interventions, focusing on those who are residing in the most at-risk areas and/or most vulnerable.

## Materials and methods

### Study area

**Fig 1:**
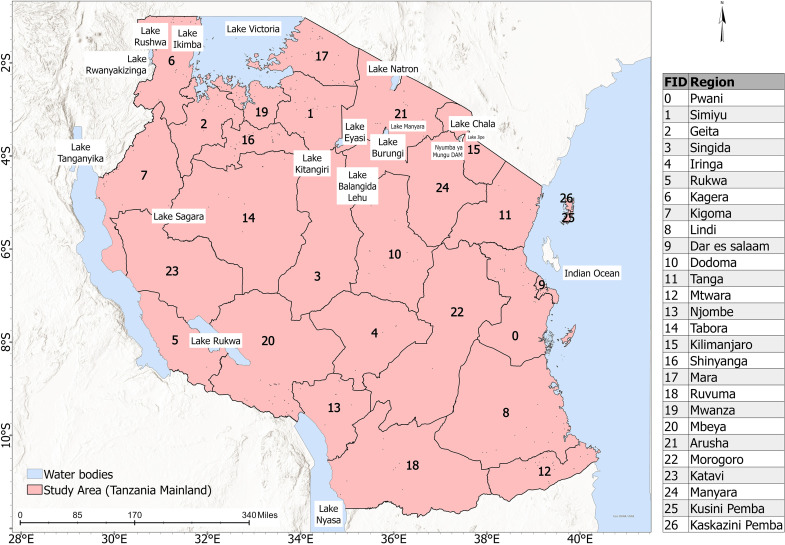
Map of Tanzania.

The United Republic of Tanzania is a country located in East Africa (Fig 1). Geographically, it is located at 6.3690^◦^ S, and longitude of 34.8888^◦^ E, bordering Uganda, Kenya, the Indian Ocean, Mozambique, Malawi, Zambia, Rwanda, Burundi, and the Democratic Republic of Congo. Spatially, Tanzania covers a land area of about 945,087 km^2^. Composed of 30 administrative units, the population of Tanzania is about 62 million [30,31].

Tanzania is divided into four main climatic zones; the humid coastal plain; the semi-arid zone of the central plateau; the high-most lake regions; and the temperate highlands area [31]. In terms of temperature, the average temperature in Tanzania ranges between 27 degrees to 29 degrees along the coast and in the offshore islands, while temperatures range between 20–30 in the central, northern and western parts [32]. The average annual rainfall is 600–800 mm, with long rains occurring between March and May and shorter rains from October to December in the northern part of the country, with the rest of the country experiencing rain from December to May [22,33]. In the central part of Tanzania, the annual rainfall is approximately 550mm, while in the south-western highlands, the annual rainfall is about 3690 mm [32].

As a result of climate change, there will likely be changes and variability in temperature and precipitation. For example, in the 2050s the average annual temperature is expected to increase by 1–3 degrees [34]. Further, precipitation is projected to become more unpredictable, with changes in rainfall quantity and the onset of the rainy season(s) [35]. Indeed, research has shown that while the interior regions will see a decrease in precipitation by up to 20%, increasing the risk of drought, other areas of Tanzania will see an increase in rainfall, increasing the severity and frequency of floods [22,36].

### Description of data

This paper is based on secondary data collected as part of the Demographic Health Survey (DHS) Program with support from the United States Agency for International Development (USAID). The data was acquired following a written request. We used the DHS Geospatial Covariates data and GPS locations cluster data (n = 628) from the spatial data repository. GPS locations of clusters are recorded during data collection in the field, and verified to ensure that they are within the right administrative boundaries in Tanzania [37]. However, to protect the confidentiality and privacy of the study participants (i.e., people showing clinical symptoms of *Plasmodium falciparum* malaria), the DHS cluster GPS locations are geomasked by displacing the cluster by up to 2 km and up to 10 km for urban and rural clusters, respectively [37]. The geomasked points do not fall outside the administrative boundary of their associated clusters [38]. A detailed description of the DHS Geospatial Covariate and GPS data can be found in [37]. The DHS data adheres to the ethical standards of the ICF Institutional Review Board and country-specific (i.e., Tanzania) ethical guidelines. Participants’ confidentiality was ensured by displacing survey coordinates. Informed consent was read to each participant, and only participants who consented proceeded to answer the survey questions.

### Measures

The outcome variable is the annual average clinical cases of *P. falciparum* malaria incidence in a DHS cluster location. Clinical cases of *P. falciparum* malaria can be described as malaria-attributable febrile episodes (i.e., a person’s body temperature beyond 37.5 C), which usually comes with nausea, fatigue, headaches, sweating and censored by a 30-day period. The occurrence of multiple sessions of these systems within the same 30-day period is classified as a single episode [37].

To explore the spatio-temporal association between the environment and *P. falciparum* malaria incidence, we used 9 environmental factors including aridity, temperature, rainfall, the number of wet days, population, Enhanced Vegetation Index (EVI), Potential Evapotranspiration (PET), precipitation and the use of Insecticide Treated Nets (ITN). Table 1 shows the detailed description of these variables.

**Table 1:** Description of the environmental covariates.

Covariates	Description
Aridity	The Aridity Index (AI) is computed by dividing the average yearly precipitation by the average yearly potential evapotranspiration as outlined by the United Nations Environmental Programme (UNEP). The index is between 0 (most arid) and 300 (most wet).
Enhanced Vegetation Index (EVI)	This is the average vegetation index within 2 km in urban clusters and 10 km in rural clusters. EVI are produced globally from MODIS sensor onboard the Terra satellite (MOD13A3). EVI products are computed from atmospherically corrected bi‐directional surface reflectance data that have been masked for water, clouds, heavy aerosols, and cloud shadows.
Insecticide Treated Nets (ITN)	ITN coverage refers to the average number within 2km in urban areas and 10 km in rural areas surrounding the survey cluster who slept in a treated insecticide net the night preceding the survey. The use of insecticide nets serves as a physical barrier that mediates human exposure to anopheles mosquitoes.
Population	Refers to the total number of people residing within a 5 x 5km pixel in the cluster location at the time of measurement. The estimation of subnational population used here is based on WorldPop project by the University of Southampton. The methodology can be accessed in [39].
Potential Evapotranpiration (PET)	PET is the mean potential evapotranspiration in a DHS cluster location in millimeters. This variable is generated by averaging the 12 months data for each given year. PET is a synthetic measurement computed using an adaptation of the Penman-Montieth formula. For the full formular, see [40]. The computation is based on variables such as mean, maximum and minimum temperature as well as vapor pressure, and cloud cover surfaces. The number generated indicates the millimeters of water that would be evaporated in a year, assuming an unlimited water in the cluster location.
Precipitation	This refers to the measured average monthly precipitation at each DHS cluster location in each year in millimeters. The dataset for 12 months is averaged to produce the mean precipitation of the year for each cluster.
Rainfall	The average annual rainfall within the 2 km (urban) or 10 km (rural) buffer surrounding the DHS survey cluster location.
Temperature	Refers to the mean annual daytime land surface temperature within the 2 km (urban) or 10 km (rural) buffer surrounding the DHS survey cluster location obtained from satellite imagery. LST is theoretically and empirically different from temperatures that are measured using ground stations. Temperatures are in degree Celsius at a spatial resolution of 0.05^o^ X 0.05^o^ produced using Moderate Resolution Imaging Spectroradiometer (MODIS) product (MYD11C3).
Wet days	This refers to the average number of days per month that received precipitation greater than 0.1mm in a cluster location each year. This measure is computed using the observed days with rainfall from meteorological stations and the number of days with expected rainfall using a formula by [40].

**Source:** Mayala, Benjamin, Thomas D. Fish, David Eitelberg, and Trinadh Dontamsetti. 2022. *The DHS Program Geospatial Covariate Datasets Manual* (Third Edition). Rockville, Maryland, USA: ICF

### Analytical approach

The analysis included preprocessing the data, such as linking the geospatial covariate cluster data to the GPS cluster points and subsequently linking them to the Tanzania district boundaries. The coordinates of the cluster were projected from the WGS 1984 Geographic Coordinates System to the Arc 1960/ UTM zone 35S (EPSG:21035 with transformation: 1122) for analysis. The data analysis was implemented in R Studio version 2023.12.1 Build 402 and ArcGIS Pro version 3.2.0. The analysis included descriptive statistics, the use of choropleth maps, hotspot analysis (Getis-Ord Gi*), Ordinary Least Square (OLS) regression, and Multiscale Geographically Weighted Regression (MGWR).

#### Hotspots and cold spots analysis (Getis-Ord Gi*).

The Getis-Ord Gi* hotspots and cold spots analysis was used to map spatial clusters of high and low values of *P. falciparum* malaria incidence in Tanzania using a fixed distance band. A fixed distance band computes each feature considering the neighbouring features with use of a binary spatial weighting system [41]. Neighbouring features within the Euclidean distance are assigned a weight of 1, and features outside the Euclidean distance are assigned a weight of 0. In this paper, we utilized the Getis-Ord Gi* to compute output classes with z-scores, p-values and confidence intervals (i.e., 99%, 95% and 90% confidence intervals). Higher and positive z-scores depict statistically significant *P. falciparum* hotspots while negative and low z-scores indicate statistically significant cold spots of *P. falciparum*. The absolute value of the z-score depicts the intensity of clustering. Getis-Ord Gi* analysis identifies different intensities of clustering at various confidence levels. The Getis-Ord* analysis is computed as follows.

$Gi*=\frac{\sum_{i=1}^{n}w_{i,j}x_{j}-X\sum_{j=1}^{n}w_{i,j}}{\frac{S\sqrt{\left[n\sum_{j=1}^{n}w_{i,j}-{\left(\sum_{j=1}^{n}w_{i,j}\right)}^{2}\right]}}{n-1}}$ (1)

$X=\frac{\sum_{j=1}^{n}w_{i,j}}{n}\;$ (2)

$S=\sqrt{\frac{\sum_{j=1}^{n}x^{2}j}{2}}-(X{)}^{2}\;$ (3)

Where,

Gi* is the spatial autocorrelation statistic of an event i over n events.

Wj = spatial weight between i and j

n = is the total number of data points

xj = characterizes the magnitude of the variable x at events j over all n

S = standard deviation

#### Multiple ordinary least square (ols) regression.

The multiple OLS regression was used as a global model to assess the linear relationship between predictors and *P. falciparum* incidence in Tanzania. OLS is a global model that utilizes a linear equation in assessing the association between a linear outcome (e.g., malaria incidence) and a set of explanatory factors. OLS assumes a stationary relationship between the outcome variables and predictors, the result of which is the generation of a single coefficient for each variable [42]. The OLS regression is mathematically expressed as

$y=\beta_{0}+\beta_{1}x_{1}+\beta_{2}x_{2}+\ldots \beta_{n}x_{n}+\varepsilon \;$ (4)

Where;

y = the response variabley = the response variable (i.e., *P. falciparum* incidence)

*x*_n_ = explanatory variables (aridity, ITN, EVI, PET, temperature, precipitation, rainfall, population, wet days)

β_0_ = Intercept

β_1_ = Parameter estimate of explanatory variable one

ɛ = Standard error

#### Multiscale Geographically Weighted Regression (MGWR).

Traditional global regression modeling such as OLS assumes that the association between variables are constant across a study area (e.g., Tanzania). However, spatial processes such as environmental factors may vary across geographic contexts, thus the use of global regression models will lead to misspecification because of the application of a constant value across all locations in the study area [43]. In GWR, spatial weights are assigned to the regression coefficient to generate different local coefficients across locations [44]. In GWR, a local regression equation is generated for each spatial unit, this allows spatial variation of the regression coefficients in the study location. The GWR can be described mathematically as follows. Given n observations, for the observation *i* ∊ {1,2,…,n} at locations *i* (*u*_*i*_, *v*_*i*_), the GWR model is given as

$y_{i}=\sum_{j=0}^{m}\beta_{j}(u_{i},v_{i})x_{ik}+\varepsilon_{i}\;$(5)

Where;

β_j_ (u_i_, v_i_)x_ik_ is the jth coefficient

Ɛ_I_ is the error term, and y_I_ is the outcome variable

u _i_, v_i_ are coordinates of geographic location *i* in space

Whereas GWR restrict the local associations within each regression model to vary at the constant spatial scale, MGWR permits the conditional associations between the outcome variable and the different explanatory variables to differ at different spatial scales [45,46]. The bandwidths indicating the data-borrowing range can change within the parameter surfaces [45]. In doing so, MGWR can provide vital insights into the scale at which different environmental processes operate, making it more flexible for assessing multiscale associations by relaxing the assumption that spatially varying phenomena in a model occur at the same spatial scale [45]. The MGWR is mathematically expressed as

$y_{i}=\sum_{j=0}^{m}\beta_{bwj}(u_{i},v_{i})x_{ik}+\varepsilon_{i\;}$(6)

Where *bwj* and *β*_*bwj*_ depicts the bandwidth utilized in the calibration of the jth conditional association.

## Results

The results are organized into four main sections. The first section provides basic description (e.g., mean, standard deviation, minimum and maximum values) of the outcome and explanatory variables. The second section uses choropleth maps to display the spatial distribution of malaria incidence from 2000 to 2020. The next section shows spatial and statistically significant clusters of malaria incidence hotspots and cold spots at different confidence intervals in 2000, 2005, 2010, 2015 and 2020. The last section highlights the global and local determinants of malaria incidence using Ordinary least square, and multiscale geographically weighted regressions.

### Descriptive statistics

**Fig 2:**
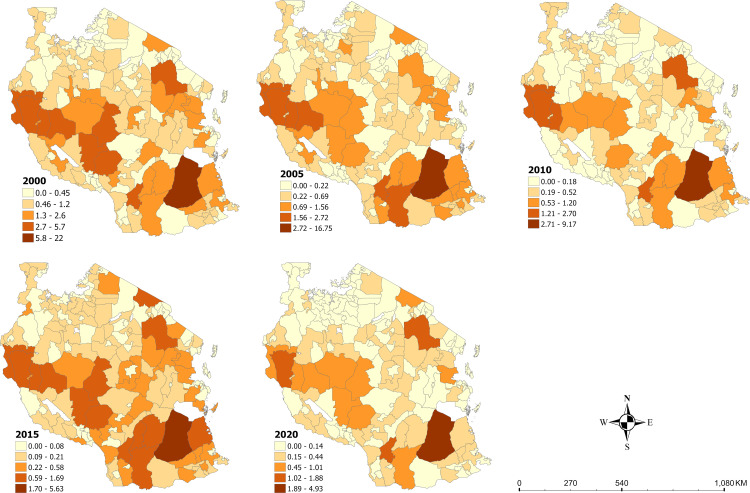
Spatial distribution of malaria incidence in Tanzania from 2000–2020.

Table 2 presents the descriptive statistics of all variables used in this paper across all the years (i.e., 2000, 2005, 2010, 2015 and 2020). The average Malaria incidence decreased from an average of 0.83 and a maximum of 22.08 in 2000 to an average of 0.18 and a maximum of 4.93 in 2020 (see Fig 2). Also, the use of insecticide treated nets increased from an average of 0.03 (maximum = 0.08) in 2000 to 0.46 (maximum = 0.87) in 2020 (see Fig 2). In 2000, the mean aridity, temperature, EVI, precipitation, rainfall, PET, Wet days, and population were 19.79, 24.31, 0.30, 72.83, 898.90, 3.71, 10.05, and 414.97, respectively. In 2020, average aridity, temperature, EVI, precipitation, rainfall, PET, Wet days, and population were 29.64, 24.07, 0.35, 108.42, 1367.70, 3.67, 12.11, and 737.24, respectively.

**Table 2:** Descriptive statistics of malaria incidence and environmental covariate from 2000 to 2020.

Environmental Covariates		Year				
		2000	2005	2010	2015	2020
**Malaria Incidence**	*Min*	0.00	0.00	0.00	0.00	0.00
	*Max*	0.57	0.57	0.45	0.28	0.31
	*Mean*	0.34	0.21	0.13	0.11	0.12
	*SD*	0.13	0.14	0.08	0.05	0.31
**ITN**	*Min*	0.01	0.09	0.19	0.05	0.04
	*Max*	0.08	0.24	0.74	0.78	0.87
	*Mean*	0.03	0.09	0.49	0.43	0.46
	*SD*	0.02	0.05	0.12	0.19	0.25
**Aridity**	*Min*	10.34	6.81	8.97	10.76	17.18
	*Max*	44.64	36.94	54.22	60.10	55.21
	*Mean*	19.79	15.89	20.36	23.55	29.64
	*SD*	5.41	5.25	6.79	7.72	6.65
**Temperature**	*Min*	15.93	16.69	16.30	16.27	16.17
	*Max*	29.49	30.99	30.26	29.68	29.14
	*Mean*	24.31	24.67	24.51	24.63	24.07
	*SD*	2.59	2.46	2.52	2.54	2.44
**EVI**	*Min*	0.13	0.14	0.16	0.16	0.20
	*Max*	0.47	0.47	0.50	0.51	0.55
	*Mean*	0.30	0.31	0.32	0.31	0.35
	*SD*	0.07	0.07	0.07	0.07	0.07
**Precipitation**	*Min*	38.46	29.35	37.50	44.42	69.12
	*Max*	139.98	122.79	175.64	179.47	181.10
	*Mean*	72.83	61.56	75.93	86.14	108.42
	*SD*	18.05	19.05	23.22	23.93	23.92
**Rainfall**	*Min*	356.42	301.86	434.82	511.67	652.53
	*Max*	1,973.26	1,787.76	2,071.99	2,649.63	3,222.84
	*Mean*	898.90	767.13	954.76	1,060.62	1,367.70
	*SD*	246.05	246.72	260.89	343.35	430.78
**PET**	*Min*	2.98	3.16	3.09	2.85	2.93
	*Max*	4.20	4.48	4.28	4.28	4.19
	*Mean*	3.71	3.90	3.76	3.70	3.67
	*SD*	0.27	0.24	0.23	0.29	0.25
**Wet days**	*Min*	7.10	6.25	7.04	7.34	9.60
	*Max*	17.17	15.77	18.96	18.16	19.05
	*Mean*	10.05	9.17	10.42	10.85	12.11
	*SD*	17.17	1.74	2.42	2.15	2.10
**Population**	*Min*	2.56	2.94	3.43	4.02	4.52
	*Max*	10,894.31	12,520.54	14,630.36	17,137.28	17,628.89
	*Mean*	414.97	476.91	557.25	652.71	737.24
	*SD*	1,173.95	1,349.19	1,576.54	1,846.69	2,021.11

Min = Minimum value, Max = Maximum value, SD = Standard Deviation

### Spatial distribution of malaria incidence from 2000 to 2020 in Tanzania

The spatial distribution of malaria incidence from 2000 to 2020 are shown using choropleth maps (Fig 2). In 2000, the Lindi region in southern Tanzania had the highest malaria incidence and consistently through 2005, 2010, 2015 and 2020. The Katavi region in West-North Tanzania, the Mbeya and Singida in central Tanzania and Manyara in Eastern Tanzania had the next highest proportion of their populations with recorded clinical cases of *P. falciparum* malaria. In 2005, parts of the Katavi and Ruvuma regions had one of the highest recorded clinical cases of *P. falciparum* malaria aside from the Lindi region. By 2020, the number of areas with high malaria incidence per population had declined aside from a few areas in the Katavi region and Manyara regions in West-North Tanzania and Eastern Tanzania, respectively. The Liwale DC in the Lindi region remained the district with the highest malaria incidence per population.

### Malaria incidence hotspots and cold spots in Tanzania from 2000 to 2020

**Fig 3:**
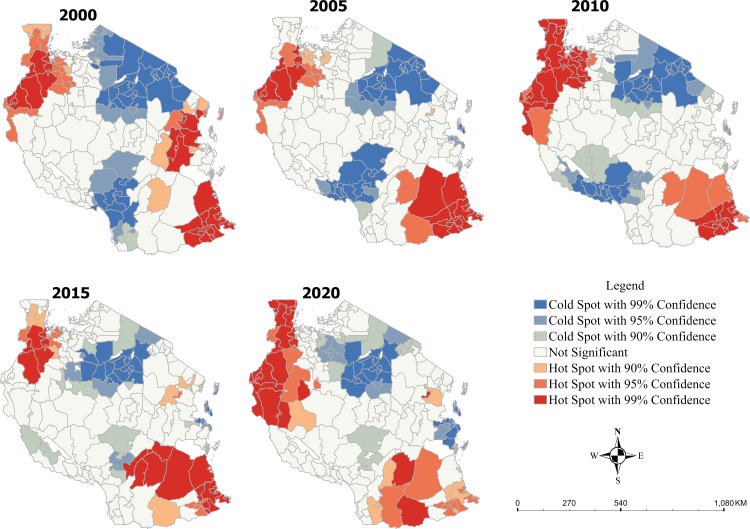
Getis-Ord Gi* hotspots analysis of malaria incidence in Tanzania from 2000–2020.

The Getis-Ord Gi* Hotspots Analysis shows statistically significant clusters of hotspots (Fig 3) and cold spots of malaria incidence at different confidence levels (e.g., 99%, 95% and 90% confidence intervals). Districts in the Kigoma and Kagera regions in the north-west of Tanzania and in the Lindi and Mtwara regions in southern Tanzania consistently had significantly higher clusters (hotspots) of malaria incidence from 2000 to 2020 at 99% confidence interval. Also, districts in the Arusha, Kilimanjaro, and parts of the Manyara regions in the north-east of Tanzania had significantly low clusters (cold spots) of malaria incidence at 95% confidence from 2000 to 2020. Districts in the Mbeya region in the south-west of Tanzania also had significantly low clusters (colds spots) of malaria incidence in 2000, 2010 and 2015 at either 99% or 95% confidence intervals.

### Environmental determinants of malaria incidence from 2000 to 2020

This section discusses the environmental determinations of malaria prevalence emphasizing first, the global level association between these environmental factors and malaria incidence using the OLS and standardized coefficients.

#### Global model results: OLS regression and predictive strength of environmental covariates.

**Fig 4:**
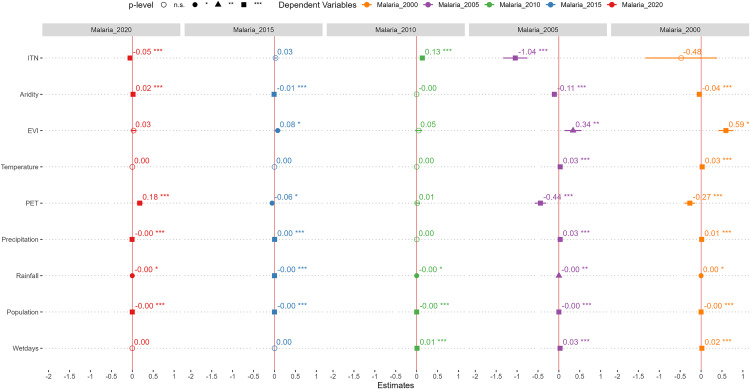
Ordinary least square (OLS) regression of the association between covariate and malaria incidence from 2000 to 2020.

The results of multiple OLS regression analysis between environmental factors and malaria incidence in Tanzania is shown in Fig 4. The results indicate that, an increase in the rate of ITN usage was significantly associated with a decrease in malaria incidence in 2020 and 2005, and inversely associated with malaria incidence in 2010. Also, an increase in aridity significantly decreased malaria incidence in 2015, 2005 and 2000. However, in 2020, aridity was positively associated with malaria incidence. EVI was significantly and positively associated with malaria incidence in 2015 and 2005. Similarly, temperature was significantly and positive related with malaria incidence in 2005 and 2000. Precipitation was also positively associated with malaria incidence in 2015, 2005 and 2000. Rainfall was significantly associated with malaria prevalence in 2020, 2015, 2010, 2005 and 2000, albeit its negligible coefficient. Similarly, as the number of wet days increased, malaria incidence increased, indicating a positive relationship.

**Fig 5:**
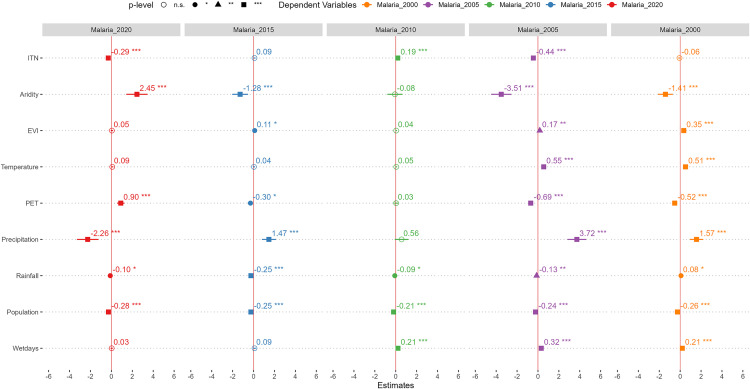
Standardized beta coefficients of the environmental covariates of *P. falciparum* from 2000 to 2020.

To understand the strength of each environmental factor in predicting *P. falciparum*, we used to standardize beta coefficients (Fig 5). Aridity (2020[β = 2.45], 2015[β = -1.28]) and precipitation (2020[β = -2.26], 2015[β=1.47]) were consistent the two strongest environmental predictors of *P. falciparum* in 2020 and 2015. In 2010, precipitation and the number of wet days were the two strongest environmental predictors of plasmodium falciparum. Also, precipitation and aridity emerged as the predictors of *P. falciparum* with the highest beta coefficients in 2005 and 2000.

#### Local model results: Multiscale geographically weighted regression (MGWR).

**Fig 6:**
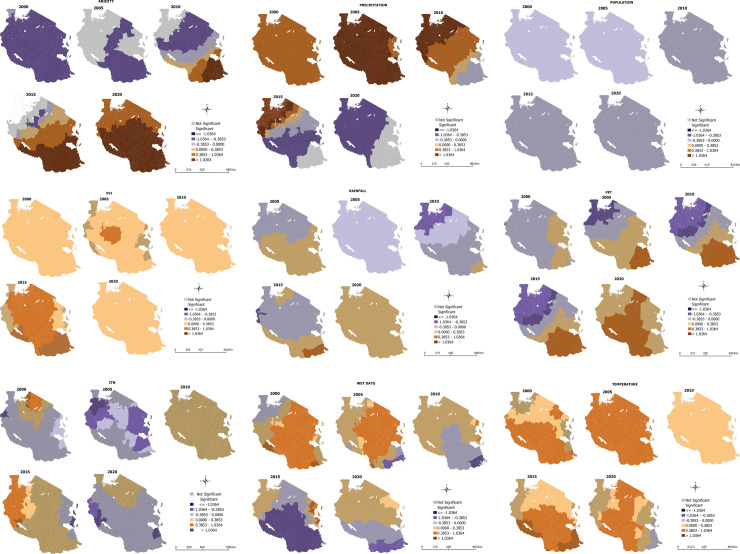
a. MGWR of Aridity and malaria incidence, b. MGWR of precipitation and malaria incidence, c. MGWR of population and malaria incidence, d. MGWR of EVI and malaria incidence, e. MGWR of Rainfall and malaria incidence, f. MGWR of PET and malaria incidence, g. MGWR of ITN usage and malaria incidence, h. MGWR of number of wet days and malaria incidence, i. MGWR of temperature and malaria incidence.

We explored the local relationships between the predictor variables and malaria incidence in Tanzania from 2000 to 2020. Results from the local MGWR is shown in Fig 6. Results from the MGWR showed no statistically significant local variation in the effect of aridity (Fig 6A) and precipitation (Fig 6B) on malaria prevalence from 2000 to 2020. Population (Fig 6C was significant and negatively associated with malaria incidence but showed no local variation in 2000 and 2005. EVI (Fig 6D) was significantly associated with malaria incidence with some local variation in 2005 and 2015 (showing a strong positive relationship in the central districts) and no local variation in 2000, 2010 and 2020.

Similarly, rainfall (Fig 6E) showed a statistically significant association with malaria in 2005 with no local variation and some local variation in 2010 (showing a strong inverse relationship in northern Tanzania and a moderate inverse relationship in central Tanzania). PET (Fig 6F) also showed a negative and statistically significant association with malaria incidence in 2010 and 2015, especially in northern Tanzania. ITN usage (Fig 6G), the number of wet days (Fig 6H), and temperature (Fig 6I) were significantly associated with malaria incidence with somewhat consistent local variation between 2000 and 2020. For example, the number of wet days had significant local variation in throughout the years. Temperature had significant local variations in the association with malaria incidence in 2000, 2015 and 2020.

**Fig 7:**
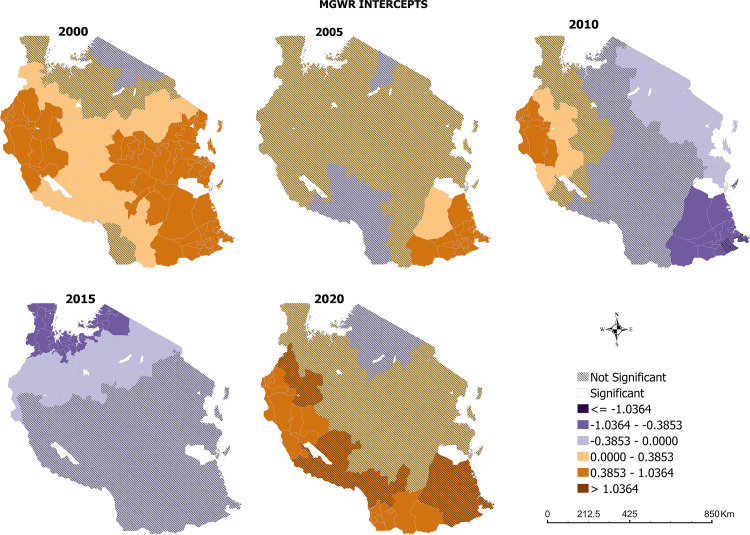
MGWR of the intercepts of malaria incidence in Tanzania.

The predicted values of malaria incidence without the influence of any environmental factor is shown in Fig 7. The results showed statistically significant and local variations in 2000, 2005, 2010, 2015 and 2020.

## Discussion

Malaria is a significant public health concern in sub-Saharan Africa (SSA), with Tanzania being one of the heavily burdened countries. Our study explores the spatial and temporal dynamics in the environmental determinants of *P. falciparum* malaria prevalence in Tanzania. Our study contributes to malaria epidemiology in Tanzania in three ways. First, we show the significant cluster of *P. falciparum* incidence in Tanzania. Second, we highlight the environmental predictors of *P. falciparum* incidence in Tanzania as well as identifying the most important environmental predictors in the last two decade. Third, we explore the local spatio-temporal variation in the association between environmental factors and *P. falciparum* incidence in Tanzania. This approach provides a comprehensive understanding of the spatial and temporal distribution of malaria and its environmental drivers to inform the development of targeted malaria control interventions and policies.

Our findings showed significant clusters of *P. falciparum* incidence in the regions of Kigoma and Kagera in the north-west of Tanzania over two decades, which is consistent with findings from [47,48]. For example, between 2007 and 2008, malaria prevalence was estimated to be 40% among children under five in the Kagera region [47]. Kagera has historically been a hotspot of malaria incidence, experiencing a severe malaria outbreak between 1997 and 1998 [47,49]. The population of Kagera is highly rural (about 94%, representing 5% of Tanzania’s population), partly explaining the high incidence of *P. falciparum* malaria incidence. In rural areas, the malaria avoidance behaviour that characterizes urban centres might not be so prevalent in rural areas, exposing populations to mosquito bites. Unlike rural housing infrastructure, urban housing structures may restrict mosquito access [50]. Furthermore, mosquito breeding is also more prevalent in areas with high agricultural land use, such as Kagera [8,51]. Similarly, our findings showed significant clusters of *P. falciparum* malaria incidence in southern Tanzania’s Lindi and Mtwara region, which concur with [3,52]. The clusters of *P. falciparum* malaria in these regions may be explained by the lack of indoor residual spraying [3] and the prevalence of biomodel rainfall, leading to high malaria transmission [53]. Moreso, the study revealed that the districts of Arusha and Kilimanjaro, and parts of the Manyara regions in the north-east of Tanzania had significantly low clusters (coldspots) of malaria incidence in the last two decades. This finding concurs with previous studies indicating that malaria prevalence was as low as 5% and 3% in northern and central parts of Tanzania, respectively compared to the southern and north-western Tanzania with malaria prevalence of about 33%-38%[3,54],

Consistent with the literature on environmental determinants of malaria in SSA [9,11, 12,16,55,56], our findings revealed that environmental factors such as temperature, aridity, landcover (i.e., vegetation), evapotranspiration, precipitation, rainfall, the use of ITN usage and the number of wet days were significantly associated with *P. falciparum* incidence in Tanzania. The use of ITN, population, PET and aridity were often adversely associated with *P. falciparum* incidence. The findings revealed that as ITN usage increased among the population, the incidence of *P. falciparum* reduced significantly in Tanzania. These findings corroborate the vital role of insecticide treated mosquito nets in SSA [16,57], with empirical evidence from more than 80 clinical trials all indicating that ITN usage prevents malaria transmission and mortalities [58]. The inverse relationship between population and malaria incidence can be explained by a higher human-to-mosquito ratio and the mosquito avoidance behaviour of urban centres with higher populations [16]. Also, higher evapotranspiration and aridity rates can reduce the level of ponds and puddles that serves as breading grounds for mosquito, thus reducing *P. falciparum* malaria incidence.

Among the environmental factors, EVI, temperature, precipitation, and the number of wet days were found to be directly related to *P. falciparum* prevalence in Tanzania. The relationship between vegetative landcover is consistent with findings from [12] that higher croplands and grasslands landcovers were associated with high malaria transmission in Tanzania. Also, increased temperatures were associated with increased *P. falciparum* prevalence, which is consistent with other studies that conclude that malaria increases with higher temperatures under favourable conditions [15,58]. Similarly, precipitation and the number of wet days were positively associated with *P. falciparum* prevalence in Tanzania. This association can be explained by the role of ponds and puddles as breeding grounds for anopheles mosquito larvae. Increased precipitation and the number of wet days may translate to available stagnant water which favours mosquito breeding. Also, our findings further reveal that the most important environmental factors that mediate malaria incidence in Tanzania in the last 2 decades were precipitation, aridity, and the number of wet days. Identifying the most vital environmental predictors of malaria incidence is a prerequisite for more targeted malaria prevention programs.

As highlighted in Nancy Krieger’s seminal work on “Epidemiology and the web of causation: Has anyone seen the spider?,” malaria epidemiology is mediated by biological, social and ecological factors, as such there was noticeable temporal (i.e., from 2000 to 2020) and spatial (i.e., across regions in Tanzania) variation in the environmental determinants of *P. falciparum* incidence. The association between ITN usage, wet days, temperature and *P. falciparum* showed significant spatial and temporal variability. For example, the association between temperature and *P. falciparum* incidence showed both temporal and spatial Variation. While temperature provides an optimal condition for mosquito larvae development, higher temperatures could also hinder malaria transmission [16]. According to Mordecai et al. [59], the optimal temperature for malaria transmission is about 25 ⁰ C, as such very higher temperatures may impede malaria larvae development. For example, higher temperatures could mean higher evapotranspiration thereby reducing stagnant water available for mosquito breeding. Similarly, while ponds and other stagnant water bodies are generally good breeding ground for mosquitoes, Mataba et al. [60] found that they might not be important factors in mediating mosquito borne diseases in certain areas of Tanzania such as the Manyara region.

While this study provides valuable insight into the spatio-temporal footprints and determinants of *P. falciparum* malaria in Tanzania, some limitations exist. We acknowledge that malaria epidemiology in SSA is complex and mediated by biological, socioeconomic, and ecological factors, all of which were not accounted for in the study due to data limitations. Moreso, the DHS data is a cluster-level dataset and does not measure individual biological, social, and economic characteristics that affect malaria transmission. Another limitation associated with the use of cluster data such as the DHS geospatial covariates data is Modifiable Areal Unit Problem (MAUP) [61]. The use of aggregated data may affect estimates at different scales [62]. To minimize MAUP, we used the smallest available administrative and geographic unit of analysis.

## Conclusion

This study reinforces the multifactorial epidemiology of malaria in SSA by examining the spatial and temporal footprints as well as the environmental determinants of *P. falciparum* incidence in Tanzania. Our findings highlight that factors such as temperature, stagnant water availability and mosquito avoidance behaviours (ITN usage) significantly mediate malaria incidence. Importantly, the role of these factors in mediating malaria incidence varies temporarily and spatially. As such effective malaria prevention and mitigation in Tanzania ought to be based on an integrated and targeted framework. The primary interventions in Tanzania used by the National Malaria Control Programme (NMCP) mainly include indoor spraying, larvicide, insecticide nets, and prompt diagnosis tests [3]. There has also been mass social behaviour change communication to increase knowledge on malaria transmission, prevention and management.

However, in geographically and ecologically diverse country such as Tanzania, implementation of malaria prevention and mitigation intervention has not been adequately targeted [3], albeit micro variation in socio-cultural and environmental factors that mediate malaria prevalence. Our findings provide policy pointers for more targeted malaria interventions. First, there is a need for proactive malaria intervention that focus on mitigating favourable environmental conditions (e.g., stagnant water) for mosquito breeding and malaria transmission while scaling factors that hinder malaria transmission such as ITN usage. For environmental factors (e.g., populations) with no significant local variations in the effects on malaria incidence, the NMCP can employ national or general malaria intervention programs to mitigate malaria incidence. However, more locally targeted malaria intervention should be employed in regions with significant clusters of malaria incidence such as Kigoma and Kagera in the north-west of Tanzania as well as the Lindi and Mtwara regions in southern Tanzania. For localized malaria interventions, the NMCP can implement targeted interventions such as indoor residual spraying (IRS) and seasonal chemoprevention (SCP) in areas with high malaria prevalence. By implementing targeted policy initiatives, malaria control and prevention can avoid the ‘one-size-fits-all’ approach that may not meet the specific needs of high-risk populations and locations.

## Appendix

### Appendix 1, MGWR and GWR diagnostics

**Table:** 

Year	2000		2005		2010		2015		2020	
	***GWR***	***MGWR***	***GWR***	***MGWR***	***GWR***	***MGWR***	***GWR***	***MGWR***	***GWR***	***MGWR***
**R-Squared**	0.8395	0.8458	0.8815	0.8548	0.7917	0.7813	0.6967	0.6956	0.6905	0.6811
**Adjusted R-Squared**	0.773	0.8178	0.8256	0.8264	0.7277	0.7449	0.5814	0.6257	0.5690	0.6275
**AICc**	314.2986	254.5534	275.2738	248.2713	333.8838	314.7086	426.5859	401.5132	432.4216	386.6188
**Sigma-Squared**	0.2263	0.1821	0.1740	0.1734	0.2719	0.2549	0.4178	0.3739	0.4301	0.3722
**Sigma-Squared MLE**	0.1605	0.1542	0.1185	0.1452	0.2083	0.2187	0.3033	0.3044	0.3095	0.3189
**Effective Degrees of Freedom**	134.0150	160.0900	128.7246	158.3390	144.8072	162.1975	137.2043	153.8543	135.9825	161.9630

## References

[1] Oladipo HJ, Tajudeen YA, Oladunjoye IO, Yusuff SI, Yusuf RO, Oluwaseyi EM, et al. Increasing challenges of malaria control in sub-Saharan Africa: priorities for public health research and policymakers. Ann Med Surg (Lond). 2022;81:104366. doi: 10.1016/j.amsu.2022.104366 36046715 PMC9421173

[2] Heuschen A-K, Lu G, Razum O, Abdul-Mumin A, Sankoh O, von Seidlein L, et al. Public health-relevant consequences of the COVID-19 pandemic on malaria in sub-Saharan Africa: a scoping review. Malar J. 2021;20(1):339. doi: 10.1186/s12936-021-03872-2 34380494 PMC8355579

[3] World Health Organization. Global trends in the burden of malaria. Vol. 1, World Malaria Report 2020. 2020. Available from: https://www.who.int/docs/default-source/malaria/world-malaria-reports/9789240015791-double-page-view.pdf?sfvrsn=2c24349d_5

[4] World Health Organization. World malaria report 2024. 2024 [cited 22 Feb 2025]. Available from: https://www.who.int/teams/global-malaria-programme/reports/world-malaria-report-2024

[5] Liheluka EA, Massawe IS, Chiduo MG, Mandara CI, Chacky F, Ndekuka L, et al. Community knowledge, attitude, practices and beliefs associated with persistence of malaria transmission in North-western and Southern regions of Tanzania. Malar J. 2023;22(1):304. doi: 10.1186/s12936-023-04738-5 37817185 PMC10563328

[6] Mmbando BP, Kamugisha ML, Lusingu JP, Francis F, Ishengoma DS, Theander TG, et al. Spatial variation and socio-economic determinants of Plasmodium falciparum infection in northeastern Tanzania. Malar J. 2011;10:145. doi: 10.1186/1475-2875-10-145 21612637 PMC3123246

[7] World Health Organization. World malaria report 2023. 2023. Available from: https://www-who-int.proxy1.lib.uwo.ca/teams/global-malaria-programme/reports/world-malaria-report-2023

[8] Mboera LEG, Makundi EA, Kitua AY. Uncertainty in malaria control in Tanzania: crossroads and challenges for future interventions. Am J Trop Med Hyg. 2007;77(6 Suppl):112–8. doi: 10.4269/ajtmh.2007.77.112 18165482

[9] Selemani M, Mrema S, Shamte A, Shabani J, Mahande MJ, Yeates K, et al. Spatial and space-time clustering of mortality due to malaria in rural Tanzania: evidence from Ifakara and Rufiji Health and Demographic Surveillance System sites. Malar J. 2015;14:369. doi: 10.1186/s12936-015-0905-y 26409483 PMC4583746

[10] Lourenço C, Tatem AJ, Atkinson PM, Cohen JM, Pindolia D, Bhavnani D, et al. Strengthening surveillance systems for malaria elimination: a global landscaping of system performance, 2015-2017. Malar J. 2019;18(1):315. doi: 10.1186/s12936-019-2960-2 31533740 PMC6751607

[11] Randell HF, Dickinson KL, Shayo EH, Mboera LEG, Kramer RA. Environmental management for malaria control: knowledge and practices in Mvomero, Tanzania. Ecohealth. 2010;7(4):507–16. doi: 10.1007/s10393-010-0343-9 20694503

[12] Mitchell CL, Ngasala B, Janko MM, Chacky F, Edwards JK, Pence BW, et al. Evaluating malaria prevalence and land cover across varying transmission intensity in Tanzania using a cross-sectional survey of school-aged children. Malar J. 2022;21(1):80. doi: 10.1186/s12936-022-04107-8 35264152 PMC8905829

[13] Oesterholt MJAM, Bousema JT, Mwerinde OK, Harris C, Lushino P, Masokoto A, et al. Spatial and temporal variation in malaria transmission in a low endemicity area in northern Tanzania. Malar J. 2006;5:98. doi: 10.1186/1475-2875-5-98 17081311 PMC1635725

[14] Yamba EI, Fink AH, Badu K, Asare EO, Tompkins AM, Amekudzi LK. Climate drivers of malaria transmission seasonality and their relative importance in Sub-Saharan Africa. Geohealth. 2023;7(2):e2022GH000698. doi: 10.1029/2022GH000698 36743738 PMC9884660

[15] Popkin Hall ZR, Seth MD, Madebe RA, Budodo R, Bakari C, Francis F, et al. Malaria species prevalence among asymptomatic individuals in four regions of Mainland Tanzania. medRxiv. 2023:2023.12.28.23300584. doi: 10.1101/2023.12.28.23300584 38234751 PMC10793544

[16] Mohammed K, Salifu MG, Batung E, Amoak D, Avoka VA, Kansanga M, et al. Spatial analysis of climatic factors and plasmodium falciparum malaria prevalence among children in Ghana. Spat Spatiotemporal Epidemiol. 2022;43:100537. doi: 10.1016/j.sste.2022.100537 36460447

[17] Zekar L, Sharman T. *Plasmodium falciparum* Malaria. StatPearls. Treasure Island (FL): StatPearls Publishing; 2024. Available from: http://www.ncbi.nlm.nih.gov/books/NBK555962/32310422

[18] Geleta G, Ketema T. Severe malaria associated with *Plasmodium falciparum* and *P. vivax* among children in Pawe Hospital, Northwest Ethiopia. Malar Res Treat. 2016;2016:1240962. doi: 10.1155/2016/1240962 27047701 PMC4800101

[19] Mafwele BJ, Lee JW. Relationships between transmission of malaria in Africa and climate factors. Sci Rep. 2022;12(1):14392. doi: 10.1038/s41598-022-18782-9 35999450 PMC9399114

[20] Beloconi A, Nyawanda BO, Bigogo G, Khagayi S, Obor D, Danquah I, et al. Malaria, climate variability, and interventions: modelling transmission dynamics. Sci Rep. 2023;13(1):7367. doi: 10.1038/s41598-023-33868-8 37147317 PMC10161998

[21] Jones AE, Wort UU, Morse AP, Hastings IM, Gagnon AS. Climate prediction of El Niño malaria epidemics in north-west Tanzania. Malar J. 2007;6:162. doi: 10.1186/1475-2875-6-162 18062817 PMC2228309

[22] Rowhani P, Lobell DB, Linderman M, Ramankutty N. Climate variability and crop production in Tanzania. Agric For Meteorol. 2011;151(4):449–60. doi: 10.1016/j.agrformet.2010.12.002

[23] World Health Organization. SDG Target 3.3 Communicable diseases. 2024 [cited 8 Apr 2024]. Available from: https://www.who.int/data/gho/data/themes/topics/sdg-target-3_3-communicable-diseases

[24] Baker RE, Mahmud AS, Miller IF, Rajeev M, Rasambainarivo F, Rice BL, et al. Infectious disease in an era of global change. Nat Rev Microbiol. 2022;20(4):193–205. doi: 10.1038/s41579-021-00639-z 34646006 PMC8513385

[25] Uwishema O, Masunga DS, Naisikye KM, Bhanji FG, Rapheal AJ, Mbwana R, et al. Impacts of environmental and climatic changes on future infectious diseases. Int J Surg. 2023;109(2):167–70. doi: 10.1097/JS9.0000000000000160 36799840 PMC10389506

[26] Carignan A, Valiquette L, Laupland KB. Impact of climate change on emerging infectious diseases: Implications for Canada. J Assoc Med Microbiol Infect Dis Can. 2019;4(2):55–9. doi: 10.3138/jammi.2018-12-10 36337740 PMC9602962

[27] Reyes R, Ahn R, Thurber K, Burke TF. Urbanization and infectious diseases: general principles, historical perspectives, and contemporary challenges. Chall Infect Dis. 2012;123–46. doi: 10.1007/978-1-4614-4496-1_4

[28] Eisenberg JNS, Desai MA, Levy K, Bates SJ, Liang S, Naumoff K, et al. Environmental determinants of infectious disease: a framework for tracking causal links and guiding public health research. Environ Health Perspect. 2007;115(8):1216–23. doi: 10.1289/ehp.9806 17687450 PMC1940110

[29] Mora C, McKenzie T, Gaw IM, Dean JM, von Hammerstein H, Knudson TA, et al. Over half of known human pathogenic diseases can be aggravated by climate change. Nat Clim Chang. 2022;12(9):869–75. doi: 10.1038/s41558-022-01426-1 35968032 PMC9362357

[30] UNICEF. Country Office Annual Report 2022: United Republic of Tanzania. 2022. Available from: https://www-unicef-org.proxy1.lib.uwo.ca/media/136491/file/United-Republic-of-Tanzania-2022-COAR.pdf

[31] Ministry of Foreign Affairs and East African Cooperation. Tanzania Country Profile. 2024 [cited 8 Apr 2024]. Available from: https://www.foreign.go.tz/tanzania/category/country-profile

[32] World Bank Group. Tanzania. In: Climate Change Knowledge Portal [Internet]. 2021 [cited 7 Apr 2024]. Available from: https://climateknowledgeportal.worldbank.org/

[33] ICID. Tanzania country profile [Online]. 2010. Available from: www.icid.org

[34] Chevallier R. Tanzania’s vulnerability to climate change impacts. South African Institute of International Affairs. 2019. pp. 8–10. Available from: https://www.jstor.org/stable/resrep29563.6

[35] Rohli RV, Ates SA, Rivera‐Monroy VH, Polito MJ, Midway SR, Castañeda‐Moya E, et al. Inter‐annual hydroclimatic variability in coastal Tanzania. Intl J Climatol. 2019;39(12):4736–50. doi: 10.1002/joc.6103

[36] Paavola J. Livelihoods, vulnerability and adaptation to climate change in Morogoro, Tanzania. Environ Sci Policy. 2008;11(7):642–54. doi: 10.1016/j.envsci.2008.06.002

[37] Mayala B, Fish TD, Eitelberg D, Dontamsetti T. The DHS program geospatial covariate datasets manual third edition the DHS program, icf 2. 2022.

[38] Burgert CR, Colston J, Roy T, Zachary B. Geographic displacement procedure and georeferenced data release policy for the Demographic and Health Surveys. ICF International; 2013.

[39] Lloyd CT, Chamberlain H, Kerr D, Yetman G, Pistolesi L, Stevens FR, et al. Global spatio-temporally harmonised datasets for producing high-resolution gridded population distribution datasets. Big Earth Data. 2019;3(2):108–39. doi: 10.1080/20964471.2019.1625151 31565697 PMC6743742

[40] Harris I, Osborn TJ, Jones P, Lister D. Version 4 of the CRU TS monthly high-resolution gridded multivariate climate dataset. Sci Data. 2020;7(1):109. doi: 10.1038/s41597-020-0453-3 32246091 PMC7125108

[41] Getis A, Ord JK. The analysis of spatial association by use of distance statistics. Geogr Anal. 1992;24(3):189–206. doi: 10.1111/j.1538-4632.1992.tb00261.x

[42] Puntanen S, Styan GPH. The Equality of the ordinary least squares estimator and the best linear unbiased estimator. Am Stat. 1989;43(3):153–61. doi: 10.1080/00031305.1989.10475644

[43] Oshan TM, Smith JP, Fotheringham AS. Targeting the spatial context of obesity determinants via multiscale geographically weighted regression. Int J Health Geogr. 2020;19(1):11. doi: 10.1186/s12942-020-00204-6 32248807 PMC7132879

[44] Brunsdon C, Fotheringham AS, Charlton ME. Geographically weighted regression: a method for exploring spatial nonstationarity. Geogr Anal. 1996;28(4):281–98. doi: 10.1111/j.1538-4632.1996.tb00936.x

[45] Fotheringham AS, Yang W, Kang W. Multiscale geographically weighted regression (MGWR). Ann Am Assoc Geogr. 2017;107(6):1247–65. doi: 10.1080/24694452.2017.1352480

[46] Yang W. An extension of geographically weighted regression with flexible bandwidths. Thesis, University of St Andrews. 2014. Available from: https://research-repository.st-andrews.ac.uk/handle/10023/7052

[47] West PA, Protopopoff N, Rowland M, Cumming E, Rand A, Drakeley C, et al. Malaria risk factors in North West Tanzania: the effect of spraying, nets and wealth. PLoS One. 2013;8(6):e65787. doi: 10.1371/journal.pone.0065787 23762425 PMC3676352

[48] Mosha JF, Lukole E, Charlwood JD, Wright A, Rowland M, Bullock O, et al. Risk factors for malaria infection prevalence and household vector density between mass distribution campaigns of long-lasting insecticidal nets in North-western Tanzania. Malar J. 2020;19(1):297. doi: 10.1186/s12936-020-03369-4 32819368 PMC7441624

[49] Carlstedt A. Malaria catastrophe in east Africa. Lancet. 1997;350(9085):1180. doi: 10.1016/S0140-6736(05)63830-5 9343534

[50] Larson PS, Eisenberg JNS, Berrocal VJ, Mathanga DP, Wilson ML. An urban-to-rural continuum of malaria risk: new analytic approaches characterize patterns in Malawi. Malar J. 2021;20(1):418. doi: 10.1186/s12936-021-03950-5 34689786 PMC8543962

[51] Aikambe JN, Mnyone LL. Retrospective analysis of malaria cases in a potentially high endemic area of Morogoro Rural District, Eastern Tanzania. Res Rep Trop Med. 2020;11:37–44. doi: 10.2147/RRTM.S254577 32607048 PMC7297450

[52] Baraka V, Ishengoma DS, Fransis F, Minja DTR, Madebe RA, Ngatunga D, et al. High-level *Plasmodium falciparum* sulfadoxine-pyrimethamine resistance with the concomitant occurrence of septuple haplotype in Tanzania. Malar J. 2015;14:439. doi: 10.1186/s12936-015-0977-8 26542942 PMC4635599

[53] Kitojo C, Chacky F, Kigadye ES, Mugasa JP, Lusasi A, Mohamed A, et al. Evaluation of a single screen and treat strategy to detect asymptomatic malaria among pregnant women from selected health facilities in Lindi region, Tanzania. Malar J. 2020;19(1):438. doi: 10.1186/s12936-020-03513-0 33256758 PMC7708125

[54] Chacky F, Runge M, Rumisha SF, Machafuko P, Chaki P, Massaga JJ, et al. Nationwide school malaria parasitaemia survey in public primary schools, the United Republic of Tanzania. Malar J. 2018;17(1):452. doi: 10.1186/s12936-018-2601-1 30518365 PMC6280377

[55] Jones AE, Wort UU, Morse AP, Hastings IM, Gagnon AS. Climate prediction of El Niño malaria epidemics in north-west Tanzania. Malar J. 2007;6:162. doi: 10.1186/1475-2875-6-162 18062817 PMC2228309

[56] Thomson MC, Ukawuba I, Hershey CL, Bennett A, Ceccato P, Lyon B, et al. Using rainfall and temperature data in the evaluation of national malaria control programs in Africa. Am J Trop Med Hyg. 2017;97(3_Suppl):32–45. doi: 10.4269/ajtmh.16-0696 28990912 PMC5619931

[57] WHO. World malaria report 2020. 2020 [cited 13 May 2021]. Available from: https://www.who.int/publications-detail-redirect/9789240015791

[58] Lengeler C. Insecticide-treated nets for malaria control: real gains. Bull World Health Organ. 2004;82(2):84. 15042228 PMC2585896

[59] Mordecai EA, Paaijmans KP, Johnson LR, Balzer C, Ben-Horin T, de Moor E, et al. Optimal temperature for malaria transmission is dramatically lower than previously predicted. Ecol Lett. 2013;16(1):22–30. doi: 10.1111/ele.12015 23050931

[60] Mataba GR, Kafula YA, Mwaijengo GN, Snoeks JM, Munishi L, Brendonck L, et al. Keep your natural enemies close - native predators can maintain low mosquito densities in temporary ponds in a malaria expansion area in Northern Tanzania. Sci Total Environ. 2021;794:148606. doi: 10.1016/j.scitotenv.2021.148606 34225145

[61] Rogerson PA, Fotheringham AS. The SAGE Handbook of Spatial Analysis. 2008; 1–528.

[62] Openshaw S. Ecological fallacies and the analysis of areal census data. Environ Plan A. 1984;16(1):17–31. doi: 10.1068/a160017 12265900

